# Complicated Breast Cellulitis Status Post-Bilateral Mastectomies Caused by Infection with *Staphylococcus Coagulans*

**Published:** 2025-07-20

**Authors:** Edwin Kamau, Lindsey L. O'Neal, Kitjawat Tatdanai, Casey W. Harless, Violet Nxedhlana, John L. MacArthur, Jamie L Dombach, Terri Carlson

**Affiliations:** Tripler Army Medical Center, Honolulu, HI: LTC Kamau, MAJ O'Neal, Mr. Tatdanai, MAJ MacArthur, CPT Dombach, LTC Carlson; Medical Technology Department, University of Hawai'i, Mānoa: Dr. Nxedhlana; Multidrug Resistant Organism Repository and Surveillance Network (MRSN), Walter Reed Army Institute of Research, Silver Spring, MD: Dr. Harless

*Staphylococcus schleiferi* is an opportunistic pathogen primarily associated with veterinary infections, such as otitis externa and pyoderma, in both dogs and cats. ^1^ In humans, *S. schleiferi* is a relatively rare cause of nosocomial infections such as bacteremia, endocarditis, wound and surgical site infections, and infections related to medical devices. ^2 - 4^ Studies have suggested that humans may acquire this organism through contact with dogs, ^1 , 5 , 6^ but thus far, there is no molecular evidence to confirm this.

Recent comparative genomic analysis taxonomically separated *S. schleiferi* subspecies— *S. schleiferi* subsp. *schleiferi* and *S. schleiferi* subsp. *coagulans* —into 2 species— *S. schleiferi* and *S. coagulans* —with genome phylogeny distinguishing them into 2 monophyletic clusters. ^6^ *Staphylococcus schleiferi* isolates mostly originate from humans, while *S. coagulans* isolates are found in both animals and humans. Additionally, the subspecies can be distinguished by unique features. The sialidase B gene ( *nanB* ), has been shown to be a unique marker for *S. schleiferi* , whereas the *chrA* gene is exclusive to *S. coagulans* . ^6^

This case study presents a unique instance of *S. coagulans* infection in a 63-year-old female with a history of breast cancer and implant reconstruction for almost 2 decades, who presented with a *S. coagulans* infection of the breast implant. This infection was suspected to have originated from her pet dog, but could not be molecularly proven, nor was there an obvious route of infection. This case highlights the clinical challenges and management strategies involved with the treatment of this *S. coagulans* infection.

## Case Presentation

A 63-year-old female with a history of right breast cancer, post-bilateral mastectomies with implant reconstruction 19 years earlier, presented to the emergency department with worsening right breast infection. She had completed a 10-day course of trimethoprim / sulfamethoxazole (bactrim) but continued to report small amount of purulent drainage and redness. She denied experiencing fever, chills, night sweats, nausea, vomiting, abdominal pain, or other complaints. Her vital signs were stable, and laboratory results showed white blood cell (WBC) count, platelets, and neutrophil levels within normal ranges.

A physical examination revealed a small amount of pericapsular fluid near the right breast implant. The patient was referred to plastic surgery, and the right implant was surgically removed by a covering surgeon the following day. Intra-operatively, the patient was noted by report to have extremely thin skin with some compromise, raising concerns about potential skin flap necrosis if capsulectomy were performed, so it was left in place and the incision closed over a drain. A swab specimen was sent to the microbiology laboratory for analysis.

**FIGURE 1.**
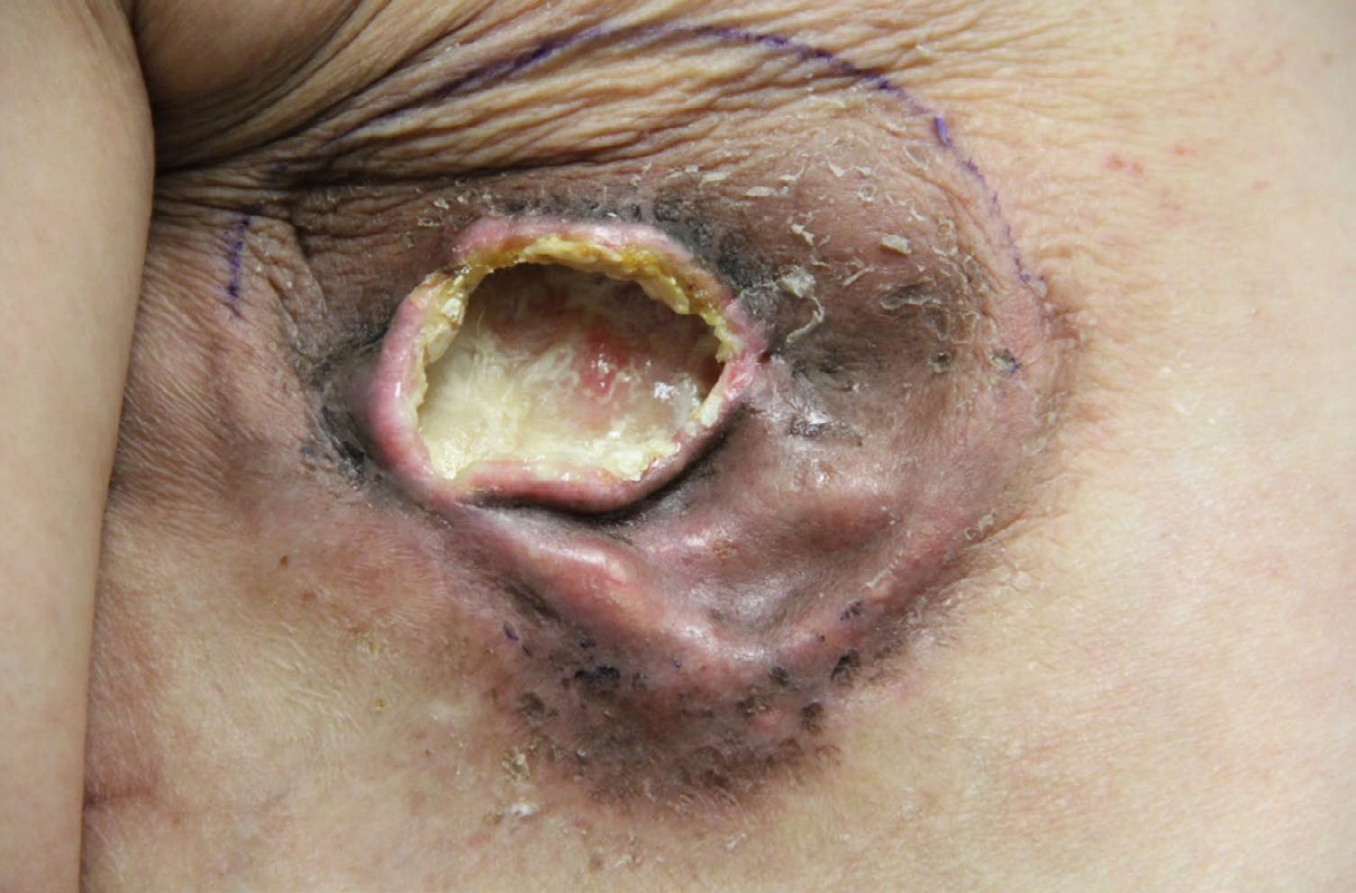
Image of the Wound, Day 12 Post-Operation, Showing Evidence of Purulent Drainage and Gaping Dehiscence

Patient care was transferred from the covering surgeon and was seen on post-operative day 12, when she exhibited purulent drainage and wound dehiscence, prompting the wound to be fully opened for adequate drainage in the clinic, as she refused admission and surgical washout **(Figure 1)** . Fluid was sent to the microbiology laboratory for analysis. Surgical debridement and coverage with a latissimus dorsi flap were recommended, but the patient continued to refuse recommended treatment, so local wound care was continued.

On post-operative day 40, the patient finally agreed to limited surgery and underwent right breast capsulectomy, wound debridement, application of a bilayer wound matrix, and placement of negative pressure wound therapy device. A tissue sample was sent to the microbiology laboratory for further analysis.

The microbiology laboratory identified all isolates from the patient as *S. schleiferi* based on MALDI-TOF (matrix-assisted laser desorption / ionization time-of-flight) analysis, and all isolates were susceptible to all antibiotics tested. The patient was treated with multiple antibiotics, including trimethoprim / sulfamethoxazole, ciprofloxacin, clindamycin, and levofloxacin. She underwent split-thickness skin grafting to achieve wound coverage and at last follow-up 6 weeks later was doing well.

For additional analysis, the isolates from the patient were whole genome sequenced (WGS) on an Illumina MiSeq or NextSeq benchtop sequencer (Illumina, Inc., San Diego, CA), as previously described, ^7^ and sequence analysis was performed using CLC Genomics Workbench (QIAGEN, Germantown, MD). The patient was also asked to swab her pet dog, and bacterial isolates from the dog identified as *S. schleiferi* by MALDI-TOF were also sequenced.

**FIGURE 2.**
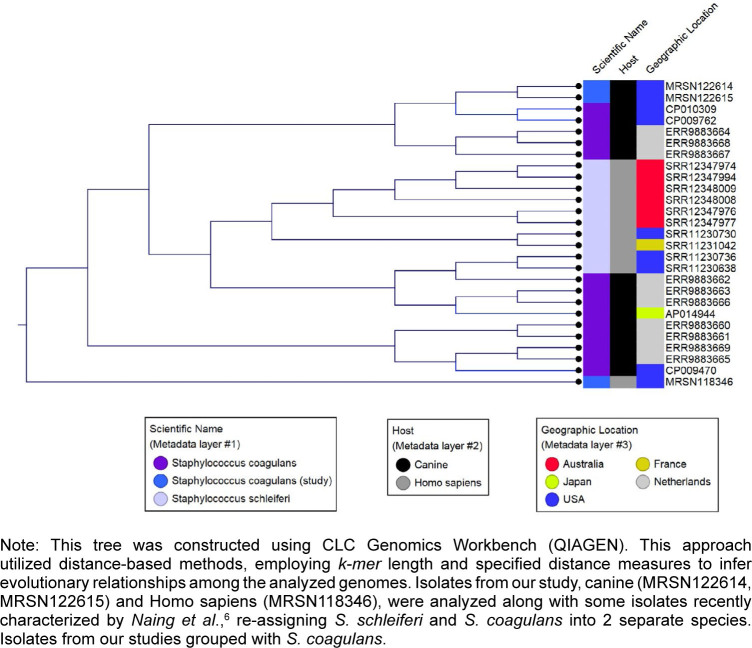
*K-mer* -based Phylogenetic Tree Showing *S. schleiferi* and *S. coagulans* Diversity and Relatedness

Based on the initial *k-mer* analysis, the isolates from both the patient and dog closely matched the reference genome CP009762 ( https://www.ncbi.nlm.nih.gov/nuccore/CP009762 ), a canine clinical isolate published in 2015. The patient isolate shared an average nucleotide identity (ANI) of 98.87% with CP009762, while the dog isolates demonstrated an ANI of 99.98%. Phylogenetic analysis of previously published WGS of *S. schleiferi* from humans and *S. coagulans* from canines, along with the isolates from this study, revealed 2 distinct *S. coagulans* clusters. The isolates from the patient's dog grouped within 1 cluster, whereas the patient's isolate formed a separate *S. coagulans* cluster **(Figure 2)** . All 3 isolates from this study carried the *chrA* gene, a marker exclusive to *S. coagulans* . SNP analysis indicated that the dog isolates were closely related, with only 12 SNP differences, while the patient isolate exhibited 16,038 SNP differences, suggesting no genetic relation.

## Discussion

To our knowledge, this is the first report of a breast implant infection caused by *S. coagulans* . When the patient first presented with cellulitis, she was administered multiple antibiotics with no symptom resolution. Interestingly, the bacteria causing the infection were susceptible to the antibiotics with which the patient was treated.

Initially, when asked if she had a pet dog, the patient denied having one. At a later date, after admitting that indeed she had a pet dog, she denied close contact with the animal, nor could she recall experiencing a skin break that may have led to the infection. It is important to note that *S. coagulans* can colonize human skin, especially those in close contact with dogs. ^8^ Although we could not molecularly confirm that the patient's infection originated from the dog, it is plausible that the patient was first colonized with the bacteria or was directly infected by the dog, but several months may have passed since the bacterial inoculation occurred. Additionally, the patient collected the specimen from the dog's mouth instead of the ears, as initially requested, and the polymicrobial nature of the dog's oral flora may have lowered the chance of obtaining a strain more molecularly related to that found in the patient.

It appeared that this may have been an endogenous infection of the breast implant, which became evident following its surgical removal. For infections related to implants, cure is mostly achieved by device removal, regardless of whether the isolates are susceptible to antistaphylococcal agents. It has been shown that in surgical infections it can take up to 12 months for a *S. schleiferi* infection to appear. ^2^ It is plausible that our patient had the infection for some time, forming biofilms that protected the bacteria from her immune system and antibiotics, leading to persistent infections despite susceptibility to the antibiotics of her treatment. ^9^ This may explain why the patient's vital signs were stable and laboratory markers, including WBC, were within normal range.

A recent study that performed molecular characterization and taxonomic reassignments of the 2 separate species of *S. schleiferi* and *S. coagulans* re-assigned several publicly available reference genomes to the correct species, including CP009762, which is now *S. coagulans* . ^6^ It is important to clinically differentiate the 2 species due to differences in host preference, pathogenic potentials, antibiotic resistance profiles, and virulence factors. ^6 , 10^

*S. schleiferi* is an important human pathogen, whereas *S. coagulans* predominantly causes infections in animals, and exhibits greater resistance to antibiotics. Current diagnostic methods routinely used in clinical microbiology laboratories, including MALDI-TOF mass spectrometry, cannot reliably differentiate the 2 species. In our case study, it would have been clinically relevant to immediately provide accurate identification of *S. coagulans* versus *S. schleiferi* . Accurate identification could have significantly influenced initial empirical treatment, given the differences in the drug susceptibility profiles of the organisms. Additionally, precise identification could have provided additional information about the possible source of infection, including pet exposure.

Although MALDI-TOF mass spectrometry can be combined with biochemical property tests for routine identification of *S. coagulans* , ^11^ development of a molecular test, such as PCR, to facilitate routine differentiation of *S. coagulans* and *S. schleiferi* , is urgently needed. Sasaki *et al* . developed a multiplex PCR method for identifying coagulase-positive *Staphylococcus* species, which could distinguish several species including *S. schleiferi* . ^12^ It remains unclear, however, whether that test can differentiate *S. coagulans* and *S. schleiferi* , potentially requiring further redevelopment. Such advancements would enhance clinical outcomes by enabling targeted treatment strategies and improving infection control measures.

This case study illustrates the clinical challenges and pathogenicity of *S. coagulans* in patients with medical devices. The successful management of this infection required a multi-disciplinary approach, including surgical intervention, proper identification of the organism, and targeted antibiotic therapy. The development of a method for discriminating between the 2 species is required for routine testing.
